# Editor’s introduction to the special issue of the 6th Biomedical Linked Annotation Hackathon (BLAH6)

**DOI:** 10.5808/GI.2020.18.2.e12

**Published:** 2020-06-24

**Authors:** Jin-Dong Kim, Kevin Bretonnel Cohen, Fabio Rinaldi, Zhiyong Lu, Nigel Collier, Hyun-Seok Park

**Affiliations:** 1 Database Center for Life Science (DBCLS), Research Organization of Information and Systems (ROIS), Kashiwa, Chiba 277-0871, Japan; 2 School of Medicine, University of Colorado, Aurora, CO 80045, USA; 3 Dalle Molle Institute for Artificial Intelligence Research (IDSIA), 6928 Manno, Switzerland; 4 National Center for Biotechnology Information (NCBI), National Institutes of Health (NIH), Bethesda, MD 20894, USA; 5 Faculty of Modern & Medieval Languages, University of Cambridge, Cambridge CB3 9 DP, UK; 6 Center for Convergence Research of Advanced Technologies, Ewha Womans University, Seoul 03760, Korea

As data science gains in importance and popularity, the need for accessing data in scientific literature is rapidly increasing. While structured databases are supposed to supply readily machine-readable data, unstructured contents, particularly scientific literature, are recognized as a biggest source of data with comprehensive details, e.g., experimental environments and actual observations.

Since the importance of scientific literature for data science has been widely recognized, several groups have invested to develop various text mining resources. While many of them are publicly available, interoperability of them remains a critical issue, hindering efficient use or reuse of them, particularly in mix with others.

The Biomedical Linked Annotation Hackathon (BLAH) series is annually organized to join forces of biomedical text mining for the goal to promote interoperability among text mining resources. The sixth edition of it was held in Tokyo, February 4–7, 2020, with 52 participants from 9 countries. The first day was held as a symposium to exchange and publicise the activities and ideas of the participants, and the following three days was held as a hackathon: the participants worked on implementing their ideas with collaboration with other participants.

While the main theme of the event was improving interoperability of biomedical literature mining, which include annotation datasets, tools, platforms, terminology resources, and so on, this year, “social media mining” was also explored as a special theme. Social media is recognized as a good source of raw signals on how people are thinking about what is going on in the world, which are largely missing in scientific literature. Therefore, social media mining is expected to complement literature mining.

This special issue is a collection of the reports on achievements from the hackathon, which address various issues of biomedical literature and social media mining, including document collection, automatic annotation, manual annotation, annotation platform, translation, terminology, ontology, and so on. Note that, except a few, many of the works began just before or even during the hackathon, and due to the limited time for work, they are often small-sized works, which are expected to benefit from collaboration with other participants. Readers will find that many of the articles have co-authorship with, or acknowledgment of other participants, which is a typical nature of hackathon-oriented publications.

We hope that this will be an opportunity for the readers of the journal Genomics & Informatics to get aware of the state-of-the-art activities regarding interoperability of biomedical text mining, and at the same time to observe activities of hackathons like BLAH.

